# Risk of HBV reactivation in relapsed or refractory diffuse large B-cell lymphoma patients receiving Bruton tyrosine kinase inhibitors therapy

**DOI:** 10.3389/fimmu.2022.982346

**Published:** 2022-08-31

**Authors:** Ying Ni, Lixia Gao, Yan Lu, Shiguang Ye, Lili Zhou, Wenbin Qian, Aibin Liang, Ping Li

**Affiliations:** ^1^ Department of Hematology, Tongji Hospital, Tongji University School of Medicine, Shanghai, China; ^2^ Department of Hematology and Oncology, Karamay Central Hospital, Karamay, China; ^3^ Department of Hematology, Qingpu Branch of Zhongshan Hospital Affiliated to Fudan University, Shanghai, China; ^4^ Department of Hematology, The Second Affiliated Hospital, College of Medicine, Zhejiang University, Hangzhou, Zhejiang, China

**Keywords:** HBV reactivation, diffuse large B-cell lymphoma, Bruton tyrosine kinase inhibitor, resolved HBV infection, prognosis

## Abstract

**Background:**

Bruton tyrosine kinase inhibitors (BTKis) interrupt B-cell receptor signaling and thereby could potentially reactivate hepatitis B virus (HBV). However, data about the risk for HBV reactivation (HBVr) of BTKis in relapsed or refractory diffuse large B-cell lymphoma (R/R DLBCL) patients are sparse.

**Methods:**

A total of 55 R/R DLBCL patients receiving BTKis therapy in the Tongji Hospital of Tongji University were enrolled. Patient clinical characteristics, treatment outcomes and details of HBVr were collected and analyzed, aiming to demonstrate the risk of HBVr in R/R DLBCL patients post BTKis therapy and the efficacy of BTKis in HBV-associated R/R DLBCL patients.

**Results:**

Of 55 R/R DLBCL patients treated with ibrutinib (N=38) and zanubrutinib (N=17), 4 were with chronic HBV infection (HBsAg positive), 26 with resolved HBV infection (HBsAg negative and HBcAb positive) and 25 without HBV infection (HBsAg negative and HBcAb negative). In resolved HBV infection group, 2 patients developed HBVr after the use of ibrutinib and zanubrutinib respectively. Neither of them developed HBV-related hepatitis. Our finding showed that the incidence of HBVr in resolved HBV infection group was 7.69% (95% CI, 0.9-25.1%). In this study, Overall response rate (ORR) was 70.9%. 1-year overall survival (OS) rate was 80.0%. Median progression-free survival (PFS) was 4 months (95% CI, 3-5 months). In addition, HBV infection was not associated with response rates or survival among R/R DLBCL patients post BTKis treatments.

**Conclusion:**

Our study suggested that HBV infection do not affect the efficacy of BTKis’ treatment. However, R/R DLBCL patients with resolved HBV infection are at a moderate risk of developing HBVr throughout BTKis treatment. Patients should be screened for HBVr during BTKis therapy.

## Introduction

Hepatitis B virus (HBV) infection has been confirmed to relate to the development of diffuse large B-cell lymphoma (DLBCL) at genomic and transcriptomic levels (1). DLBCL patients with concomitant HBV infection have a risk of HBV reactivation (HBVr) when receiving immunotherapy, targeted therapy, chimeric antigen receptor T cell therapy or cytotoxic chemotherapy (2–6). Host status, HBV status at baseline and type of therapy together determine the risk of HBVr (7). After reactivation, clinical manifestations could be totally different. Patients might be asymptomatic, experience hepatitis flares or even develop life-threatening liver failure despite discontinued from treatments (8).

Bruton tyrosine kinase inhibitors (BTKis) are proved to be tolerable and effective in relapsed or refractory DLBCL (R/R DLBCL) (9–11). Among a series of BTKis, ibrutinib is the first-in-class, and zanubrutinib is one of second-generation (12). These agents chould form an irreversible covalent bond at Cys481 within the adenosine triphosphate binding pocket of Bruton tyrosine kinase (BTK), and thereby inhibit BTK, a key component of the B-cell receptor (BCR) signaling pathway (13, 14). As interrupting BCR, which is critical in normal B lymphopoiesis, BTKis could potentially reactivate HBV (8). However, published data about the risk of HBVr induced by BTKis are limited.

Herein, we designed a retrospective study to systematically assess the HBVr rate in R/R DLBCL patients post BTKis therapy in a real world, and explore the efficacy of BTKis in HBV-associated R/R DLBCL patients. To the best of our knowledge, this is the first and largest research to evaluate the HBVr incidence induced by BTKis and the efficacy of BTKis in HBV-associated R/R DLBCL patients. Our research could help physicians to better select R/R DLBCL patients suitable for such a targeted treatment.

## Materials and methods

### Patients

This retrospective study enrolled 55 R/R DLBCL patients treated with BTKis at Tongji Hospital of Tongji University from January 2018 to April 2021. Patients were diagnosed with DLBCL by pathology according to 2016 World Health Organization classification for tumors of hematopoietic and lymphoid tissues (15). Relapsed DLBCL was defined as any new lesion or increase by 50% of previously involved sites from nadir (16). Refractory DLBCL was defined as progressive disease or stable disease as best response at any point during chemotherapy or relapsed at ≤12 months from autologous stem cell transplantation (17). Prophylactic nucleos(t)ide analog therapy (NAT) was administered to patients with chronic HBV infection, and patients with resolved HBV infection whose HBV DNA level rose above 100 IU/mL or who could not be able to adhere to HBV DNA monitoring regularly though HBV DNA level is normal. Informed consent was obtained from each patient, and the study adhered to the principles of the Helsinki Declaration.

### Data collection and covariates

Data were extracted from patients’ medical records. All patients were observed for HBVr during BTKis therapy. The primary end point was the occurrence of HBVr. The secondary end point was either disease progression or death of any cause. Clinical characteristics including age, sex, international prognostic index, BTKi subtype, time on BTKi, previous therapies, alanine aminotransferase (ALT), aspartate aminotransferase (AST), total bilirubin (TBIL), lactic acid dehydrogenase (LDH), HBV status (HBsAg, HBsAb, HBeAg, HBeAb, HBcAb and HBV DNA level) at baseline and antiviral prophylaxis were collected before commencing BTKi-containing treatment.

Serum HBV DNA level was measured with the lower limit of detection at 100 IU/mL. HBVr was defined as 1 of the following: >1 log increase in HBV DNA, HBV DNA-positive when previously negative, HBV DNA >2000 IU/mL if no baseline level was available, or reverse seroconversion from HBsAg-negative to -positive (18). Hepatitis flare was defined as serum ALT level >3×upper limit of normal (normal upper limit = 50 U/L in our center) or an ALT increase >100 U/L (19). HBV-related hepatitis was defined as hepatitis accompanying or following HBVr without any acute infection with other hepatitis viruses or any systemic disease (20, 21).

### Outcomes

Response evaluation was based on the International Working Group response criteria for non-Hodgkin lymphoma (NHL) (16, 22). Overall survival (OS) was calculated from date of receiving BTKi treatment until June 30, 2021 or death of any cause, and progression-free survival (PFS) was until June 30, 2021, disease progression, or death of any cause.

### Statistical analyses

Normality was assessed using the Shapiro-Wilk test. Equality of variances was assessed using Levene’s test. For normally distributed data with homogeneity of variance, differences between three groups were compared using one-way analysis of variance (ANOVA). For abnormally distributed data or data without homogeneity of variance, nonparametric Kruskal-Wallis test was used. We used Fisher’s exact test for categorical data. If overall comparison among three groups was significant, we would adjust P values for multiple comparisons using the Bonferroni method. OS and PFS were analyzed by the Kaplan–Meier method. Differences between survival curves were tested using log-rank test. We used univariate and multivariate Cox proportional hazard regression models to evaluate predictors of HBVr and OS. Factors with a significance of <0.2 by univariate analysis were included in the multivariate analyses, along with age and sex. All statistical tests were two-sided, and the threshold of significance was set at P<0.05. Analyses were performed using SPSS Version 26.0 (IBM Corp., Armonk, NY, USA) and GraphPad Prism 8 (GraphPad Software Inc., San Diego, CA, USA).

## Results

### Clinical characteristics of patients

**Figure 1:**
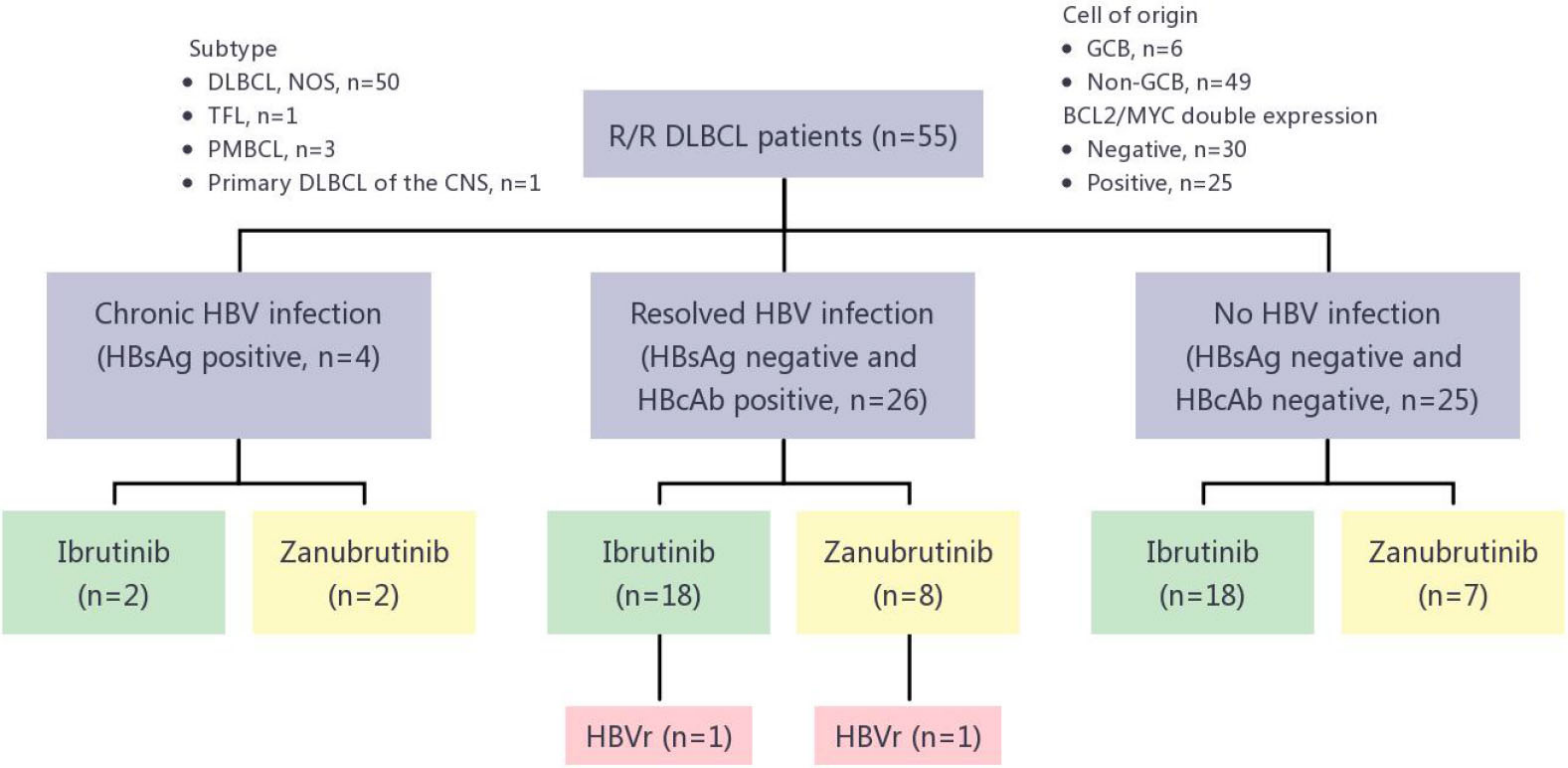
HBVr in R/R DLBCL patients treated with different BTKis. R/R DLBCL, relapsed or refractory diffuse large B-cell lymphoma; DLBCL, NOS, diffuse large B-cell lymphoma, not otherwise specified; TFL, transformed follicular lymphoma; PMBCL, primary mediastinal large B-cell lymphoma; CNS, central nervous system; GCB, germinal center B-cell–like; HBV, hepatitis B virus; HbsAg, hepatitis B surface antigen; HbcAg, hepatitis B core antibody; HBVr, hepatitis B virus reactivation; BTKis, Bruton tyrosine kinase inhibitors.

Between January 2018 and April 2021, 55 R/R DLBCL patients received BTKi-containing therapy, 4 of whom had chronic HBV infection (HBsAg positive), 26 with resolved HBV infection (HBsAg negative and anti-HBc positive) and 25 without HBV infection (**Figure 1**; **Table 1**). Median age was 58 years, ranging from 22 years to 78 years. 34 (61.8%) patients were male. 49 (89.1%) cases were non-germinal center B-cell subtype. 38 (69.1%) patients received Ibrutinib therapy, while 17 (30.9%) received zanubrutinib. The median time on BTKi was 3 months (range: 1-24 months). All 4 patients with chronic HBV infection received entecavir as prophylaxis before using BTKi. Among patients with resolved HBV infection group, 9 (34.6%) received entecavir as routine prophylaxis, while 17 patients did not use any NAT strategy. The demographic and clinical characteristics between patients in chronic HBV infection group, resolved HBV infection group and without HBV group were similar. Baseline demographics and disease characteristics for the analysis population were shown in **Figure 1** and **Table 1**.

**Table 1:** Demographics and clinical characteristics of R/R DLBCL patients enrolled in the study (N = 55) .

	Total (N = 55)	Chronic HBV infection (N = 4)	Resolved HBV infection (N = 26)	No HBV infection (N = 25)	*P-*value	*Pc*
Age (y; median; range)	58 (22–78)	53.5 (41–69)	59 (38–78)	57 (22–76)	0.628	
Male	34(61.8)	2 (50)	19 (73.1)	13 (52)	0.280	
IPI						
0-3	27 (49.1)	2 (50)	13 (50)	12 (48)	1.000	
4-5	28 (50.9)	2 (50)	13 (50)	13 (52)		
BTKi subtype						
Ibrutinib	38 (69.1)	2 (50)	18 (69.2)	18 (72)	0.734	
zanubrutinib	17 (30.9)	2 (50)	8 (30.8)	7 (28)		
Time on BTKi (mo; median; range)	3 (1-24)	3.25 (1.5-6)	3 (1-8)	4 (1-24)	0.330	
Previous therapies (median; range)	4 (1-8)	4.5 (4-8)	4 (2-6)	4 (1-7)	0.270	
ALT (U/L; median; range)	26 (5-52)	14.5 (11-25)	24 (5-52)	27 (11-40)	0.129	
AST (U/L; median; range)	25 (2-117)	27 (23-36)	23.5 (2-117)	25 (3-109)	0.441	
TBIL (μmol/L; median; range)	12 (6-72)	24 (18.5-54)	12.25 (6-72)	11 (6-72)	**0.043**	0.129
LDH(U/L;median;range)	326 (129-2150)	397.5 (174-639)	326 (166-1242)	412 (129-2150)	0.857	
Baseline HBV DNA level						
Detectable	1 (1.8)	0 (0)	1 (3.8)	0 (0)	1.000	
Undetectable	54 (98.2)	4 (100)	25 (96.2)	25 (100)		
Antiviral prophylaxis						
No	42 (76.2)	0 (0)	17 (65.4)	25 (100)	**0.000**	**0.000**
Entecavir	13 (23.6)	4 (100)	9 (34.6)	0 (0)		
Follow-up (mo; median; range)	9 (1-43)	4.5 (4-15)	7 (2-30)	10 (1-43)	0.337	

Data are presented as n (%) of patients unless indicated otherwise.

R/R DLBCL, relapsed or refractory diffuse large B-cell lymphoma; HBV, hepatitis B virus; Pc, P corrected; IPI, international prognostic index; BTKi, Bruton Tyrosine Kinase inhibitor; ALT, alanine aminotransferase; AST, aspartate aminotransferase; TBIL, total bilirubin; LDH, Lactic acid dehydrogenase.

The significant P-values were bold in this table.

### HBVr and risk factors for reactivation

**Figure 2:**
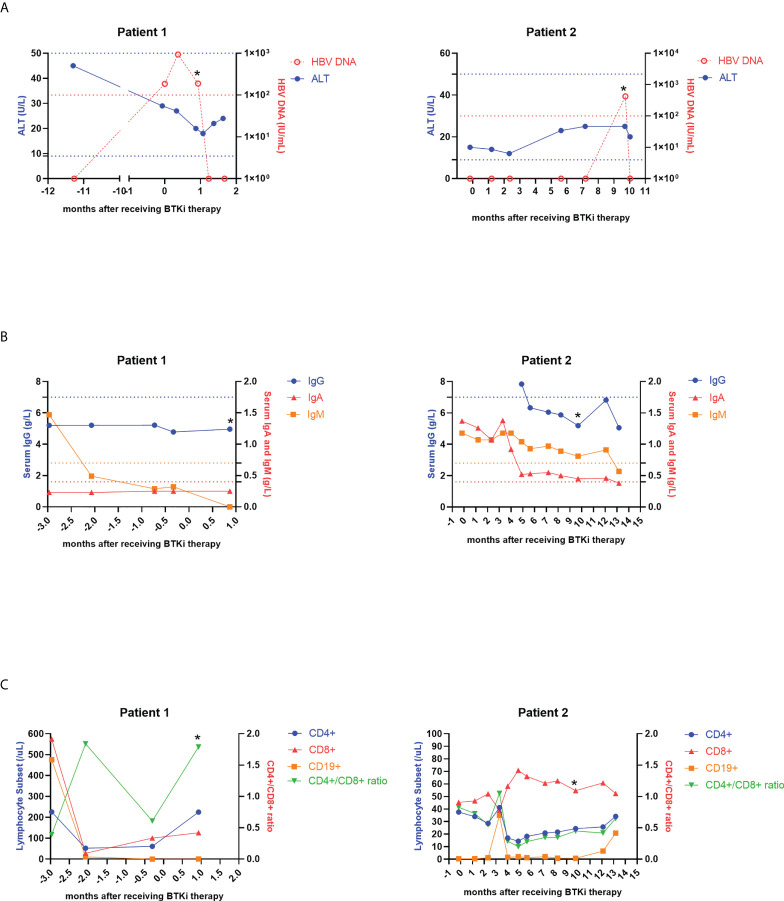
Dynamic changes of ALT, HBV DNA copies **(A)**, serum levels of IgG, IgA, IgM **(B)** and lymphocyte subsets **(C)** in two patients with HBVr. An asterisk (*) indicates HBVr. ALT, alanine aminotransferase; HBV, hepatitis B virus; BTKi, Bruton Tyrosine Kinase inhibitor; Ig, immunoglobulin; HBVr, hepatitis B virus reactivation.

Of the 26 patients with resolved HBV infection, 2 (7.69%, 95%CI, 0.9-25.1%) had HBVr (**Figure 1**). Except for these 2 patients, no patient in either chronic HBV infection group (0/4) or without HBV infection group (0/25) developed HBVr. Details for 2 patients with HBVr induced by BTKi were shown in **Table 2** and **Figure 2**.

**Table 2:** Details of 2 patients with HBVr.

	Patient 1	Patient 2
Age (y)/Sex	58/M	49/M
Ann Arbor stage	IV	IV
Group	A	B
Cell of origin	Non-GCB	Non-GCB
Refractory disease	Yes	Yes
Previous therapies	6	4
BTKi type	Ibrutinib	Zanubrutinib
Time on BTKi(mo)	1	2.5
HBV status at baseline		
HbsAg	–	–
HbsAb	+	–
HbeAg	–	–
HbeAb	–	+
HbcAb	+	+
HBV-DNA (IU/mL)	1.86×10^2^	<1×10^2^
Prophylaxis anti-virus drug	Entecavir	Entecavir
Hepatitis at baseline		
ALT (U/L)	29	15
AST (U/L)	23	16
TBIL(μmol/L)	13.9	12.4
At diagnosis of HBVr		
Time to reactivation (d)	26	292
HBV-DNA (IU/mL)	1.89×10^2^	4.21×10^2^
ALT (U/L)	18	25
AST	23	20
TBIL(μmol/L)	15.3	7.7
Response	PR	PR
PFS (mo)	1	6
OS (mo)	3	11^+^

HBVr, hepatitis B virus reactivation; M, male; non-GCB, non-germinal center B-cell–like; BTKi, Bruton tyrosine kinase inhibitor; mo, month; HBV, Hepatitis B virus; HbsAg, hepatitis B surface antigen; HbsAb, hepatitis B surface antibody; HbeAg, hepatitis B e antigen; HbeAg, hepatitis B e antibody; HbcAg, hepatitis B core antibody; ALT, alanine aminotransferase; AST, aspertate aminotransferase; TBIL, total bilirubin; d, day; PR, partial response; PFS, progression-free survival; OS, overall survival. + indicates ongoing response status.

Patient 1 was a 58-year-old male, in the advanced stage four, and had received 6 lines of treatments before ibrutinib therapy. Among these 6 regimens, the first two were rituximab-based. The seventh line therapeutic regimen was ibrutinib and rituximab combined with chemotherapy consisting of ifosfamide, cisplatin and etoposide, in which only ibrutinib is the new added agent. At baseline, HbsAg, HbeAg, HbeAb were negative, but HbsAb and HbcAb were positive. HBV-DNA at baseline was 1.86×10^2^IU/mL. He used entecavir as antiviral prophylaxis. After receiving ibrutinib-combined therapy 26 days, the patient’s HBsAg was subsequently reversed from negative to positive. Thus, he was dignosed with HBVr and ibrutinib was stopped. ALT, AST and TBIL were all normal both before using ibrutinib and at diagnosis of HBVr. No HBV-related hepatitis occurred. HBV DNA viral load at HBVr was 1.89×10^2^IU/mL, not elevated compared to baseline (**Figure 2A**). Besides, immunoglobulins and lymphocyte subsets at HBVr were not declined (**Figures 2B** **, C**). Patient 1 achieved partial remission (PR) after seventh line’s treatment, but died 3 months later.

Patient 2 was a 49-year-old male, also in the advanced stage four, and had received 4 lines of treatments previous to receiving zanubrutinib therapy. The first three were rituximab-based, and the fifth regimen was zanubrutinib combined with ESHAP, in which only zanubrutinib and cytarabine are new. At baseline, the patient was HBcAb+/HBsAb−, and HBV DNA cannot be detectable. He also used entecavir as prophylactic anti-virus drug. The patient received zanubrutinib for 2.5 months. Approximately 7.5 months after zanubrutinib therapy ended, HBV DNA of patient 2 elevated to 4.21×10^2^ IU/ml. Then we confirmed that he had developed HBVr. Hepatitis flare was also not observed in patient 2 (**Figure 2A**). Serum IgG, IgA and IgM concentrations decreased when HBV was reactivated, but lymphocyte concentrations did not change (**Figures 2B** **, C**). This patient also achieved PR, however, disease progressed 6 months later.

Univariate Cox regression analysis indicated no association between HBVr risk and sex, age, previous therapies, type of BTKi, HBsAb at baseline and HBeAb at baseline (**Table 3**). Given no independent factor significantly associated with the risk of HBVr in univariate analysis, we did not conduct multivariate analysis furthermore.

**Table 3:** Univariate Cox regression analysis for HBVr (N = 26).

	HBVr (N = 2)	No HBVr (N = 24)	HR (95% CI)	*P-*value
Female	0 (0)	7 (29.2)	0.016 (0.000-1451.874)	0.480
Age > 60 years	0 (0)	12 (50)	0.033 (0.000-62403.444)	0.644
Previous therapies≥4	2 (11.8)	15 (88.2)	0.849 (0.291-2.480)	0.765
Zanubrutinib	1 (50)	7 (29.2)	4.243 (0.231-78.065)	0.331
HBsAb-Positive	1 (50)	12 (50)	1.254 (0.077-20.400)	0.874
HBeAb-Positive	1 (50)	12 (50)	1 (0.063-15.988)	1

Comparisons in 26 patients with resolved HBV infection.

Data are presented as n (%) of patients.

HBVr, hepatitis B virus reactivation; HR, Hazard Ratio; CI, confidence interval; HbsAb, hepatitis B surface antibody; HbeAb, hepatitis B e antibody.

### Clinical responses and survival

As shown in **Table 4**, 9 patients achieved complete response and 30 patients achieved PR. Overall response rate was 70.9%. The number of patients that achieved complete response in the chronic HBVr group, resolved HBV infection group and no HBV infection group were 1 (25%), 2 (7.7%) and 6 (24%), respectively. Response rates were similar across the 3 data sets (P=0.281).

**Table 4:** Clinical response and survival of R/R DLBCL patients treated with BTKis (N = 55).

	Total (N = 55)	Chronic HBV infection (N = 4)	Resolved HBV infection (N = 26)	No HBV infection(N = 25)	*P-*value
Clinical response					
CR	9 (16.4)	1 (25)	2 (7.7)	6 (24)	0.281
PR	30 (54.5)	2 (50)	14 (53.8)	14 (56)	
SD	11 (20.0)	1 (25)	8 (30.8)	2 (8)	
PD	5 (9.1)	0(0)	2 (7.7)	3 (12)	
OS (mo; median; 95%CI)	NA	5 (3.503-6.497)	19 (NA)	NA	0.503
PFS (mo; median; 95%CI)	4 (3-5)	4 (1-7)	4 (3-5)	4 (2-6)	0.976

Data are presented as n (%) of patients unless indicated otherwise.

R/R DLBCL, relapsed or refractory diffuse large B-cell lymphoma; BTKis, Bruton tyrosine kinase inhibitors; HBV, hepatitis B virus; CR, complete response; PR, partial response; SD, stable disease; PD, progressive disease; OS, overall survival; CI, confidence interval; mo, month; NA, not applicable; PFS, progression-free survival.

**Figure 3:**
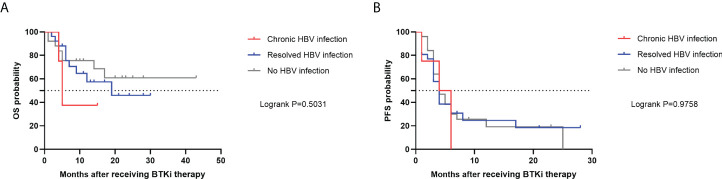
OS **(A)** and PFS **(B)** in R/R DLBCL patients after receiving BTKi therapy. OS, overall survival; PFS, progression-free survival; HBV, hepatitis B virus; BTKi, Bruton tyrosine kinase inhibitor; R/R DLBCL, relapsed or refractory diffuse large B-cell lymphoma.

Median OS was not reached based on the Kaplan-Meier analysis. 1-year OS rate was 80.0%. Although a higher OS rate among patients in the no HBV infection group was observed, the survival evaluated was similar (P=0.5031) (**Figure 3A**). PFS was consistently poor in R/R DLBCL patients, with a median PFS of 4 months (95% CI, 3-5 months). PFS rates were also similar across 3 subgroups (P=0.9758) (**Figure 3B**).

We ran univariate and multivariate Cox regression models to analyze factors influencing the patients’ mortality (**Supplementary Table 1**). International prognostic index high risk and Age ≤ 60 years were associated with inferior survival (P=0.007 and P=0.033, respectively), whereas sex, HBVr, and HBV infection did not influence OS.

## Discussion

This is the first systematic analysis of HBVr rate and BTKis’ efficacy in R/R DLBCL patients with HBV infection receiving BTKi-containing therapy. The study showed that patients with R/R DLBCL and concomitant past HBV infection do have a moderate risk of HBVr after BTKis therapy as HBVr incidence was 7.69% (95%CI, 0.9-25.1%) in our cohort. Rohit Loomba et al. summarized different classes of biologics associated with HBVr, also considering BTKi is at a moderate-risk (7). Although ibrutinib or zanubrutinib administration has a potential HBVr complication, there was no episode of HBV-hepatitis with a prophylactic NAT, and HBV infection might not affect response rates or survival among R/R DLBCL patients post BTKis treatment. In the era of targeted therapy for hematologic malignancies, it is vital for us to better estimate the risk of HBVr and optimize targeted therapeutic strategies.

In 2015, Patrick de Jésus Ngoma et al. firstly reported a case of HBVr in a 80 years old chronic lymphocytic leukaemia (CLL) patient receiving ibrutinib, revealing that in addition to hemocytopenia and some nonhematologic adverse events such as diarrhea, upper respiratory tract infection, and fatigue, ibrutinib also has a potential risk of reactivating virus (23). With widespread use in a real-world population, a few case reports and case series have reported the HBVr complication of ibrutinib administration in patients with CLL and marginal zone lymphoma (MZL) (24–27). Besides the first-generation BTKi ibrutinib, one of second-generation BTKis zanubrutinib, an highly selective BTKi, also has this infectious diseases adverse event in CLL patients (28). Herein, our study further elucidates that ibrutinib and zanubrutinib could also pose HBVr in R/R DLBCL, revealing that BTKis’ induced HBVr would not only occured in patients with indolent lymphoma, but also in aggressive lymphoma, but the incidence is slightly lower than in CLL. However, given that the size of reported samples were all small, the results must be considered preliminary and should be verified furthermore.

Of 2 patients with HBVr in our study, one case occurred 1 month after receiving BTKi, while HBVr time of another case is almost 7.5 months after BTKi therapy was ended. This phenomenon implies that similar to rituximab and PD-1, the effect of BTKis on viral reactivation would also persist beyond treatment period (29, 30). Thus, longer-term antiviral treatment is also necessary, even after cessation of BTKi treatment. High baseline HBV DNA level is a key risk factor for HBVr, and rituximab may facilitate HBV replication (3). In our study, only one case had high baseline HBV DNA level, who rapidly developed HBVr after only 1 month use of ibrutinib. However, in Cox regression analyses of our cohort, we failed to identify any factor significantly contributed to HBVr, probably because that number of HBVr cases in our study is limited. Of note, the first patient received ibrutinib therapy as the most recent and only new added agent, then HBVr occurred subsequently, supporting that BTKi do have a risk of reactivating HBV. For the second patient, the interval between the last use of rituximab and onset of HBVr was longer than 1 year, suggesting that rituximab is a less likely contributor to the HBVr. In addition, Kusumoto et al’s report about GOYA and GALLIUM demonstrated that the incidence of rituximab- or obinutuzumab-associated HBV reactivation in patients with resolved HBV infection during prophylactic NAT was only 2.1% (3). In view of the B-cell signaling inhibitory activity of BTKis, which might be more potent than rituximab in suppressing B-cells, it should be recognized that BTKis would probably increase the incidence of virus reactivation compared to rituximab.

The mechanisms underlying the increased risk of HBVr reactivation are pivotal but remain unclear. B cell dysfunction and possible T cell dysfunction might be the main cause (31, 32). Loss of BTK function results in a B-cell-dysfunction phenotype with decreased serum immunoglobulin levels and an increased predisposition to infections (33, 34). Of note, first-generation BTKi ibrutinib has off-target effects, inhibiting other tyrosine kinases, such as epidermal growth factor receptor (EGFR), JAK3, TEC, interleukin-2-inducible kinase (ITK), etc., and interacting with other important signaling pathway proteins, which might contribute to an increased risk of adverse events (35, 36). Among these kinases, ITK plays a critical role in T-cell signaling and immune regulation function. Occurrence of HBVr induced by ibrutinib might be associated with the inhibition of ITK, which results in subverting Th2 immunity and potentiating Th1-based immune responses (35, 37). In our research, one patient’s immunoglobulins decreased when HBVr occurred, indicating that humoral immunity might be inhibited by BTKi through interfering with the downstream pathways of BCR signaling. However, we need to knowledge that all patients enrolled in our study are R/R DLBCL who have received multiple lines of treatments. Thus, immunosuppressive effects of upfront and concomitant drugs could not be ignored. Different to immunoglobulins, neither of their lymphocyte concentrations changed. As the number of patients with HBVr in our study was small, the exact associations between HBVr and possible factors could hardly be analyzed. It is possible that with longer follow-up and larger cohort, the reactivation rate will be higher and more outcomes would be observed.

It has been known that PFS and survival between advanced B-cell cancers patients with chronic HBV infection cohort, with resolved HBV infection cohort and without HBV infection cohort post chimeric antigen receptor T cell therapy were similar (38, 39). In our study, response and PFS rates across above 3 data sets post BTKi therapy were also similar. However, though not statistically significant, R/R DLBCL patients with chronic HBV infection seemed to have a poorer prognosis. Additional studies are needed to explain above phenomenon.

## Conclusions

This is the first and largest research to evaluate the HBVr incidence induced by BTKis and the efficacy of BTKis in HBV-associated R/R DLBCL patients. We retrospectively collected R/R DLBCL cases receiving BTKis therapy in Tongji hospital of Tongji university, finding that HBVr rate in patients with resolved HBV infection was 7.69% (95% CI, 0.9-25.1%). Regardless of with HBV or not, response rates and survival were similar. Our research could help physicians to better select R/R DLBCL patients suitable for such a targeted treatment, and provide the deeper and comprehensive understanding that BTKis could induce HBVr in different types of B-cell NHL.

## Data availability statement

The original contributions presented in the study are included in the article/**Supplementary Material**. Further inquiries can be directed to the corresponding authors.

## Ethics statement

The studies involving human participants were reviewed and approved by the Ethics Committee of Tongji Hospital of Tongji University. The patients/participants provided their written informed consent to participate in this study. Written informed consent was obtained from the individual(s) for the publication of any potentially identifiable images or data included in this article.

## Author contributions

YN, LG, WQ, AL and PL designed the study and interpreted the data. YN performed the statistical analysis and wrote the manuscript. YN, LG, YL and SY contributed to data collection. All authors recruited patients. All authors contributed to the article and approved the submitted version.

## Funding

This work was supported by funds from the National Natural Science Foundation of China (Nos. 81830004 and 82070168), Translational Research Grant of NCRCH (2020ZKZC04), the Ministry of Science and Technology of China (2021YFA1100800) and Shanghai Municipal Health Commission (2020CXJQ02).

## Acknowledgments

We thank all people for their valuable help regarding study design and data analysis.

## Conflict of interest

The authors declare that the research was conducted in the absence of any commercial or financial relationships that could be construed as a potential conflict of interest.

## Publisher’s note

All claims expressed in this article are solely those of the authors and do not necessarily represent those of their affiliated organizations, or those of the publisher, the editors and the reviewers. Any product that may be evaluated in this article, or claim that may be made by its manufacturer, is not guaranteed or endorsed by the publisher.
