# Supplementary material for: Risk of HBV reactivation in relapsed or refractory diffuse large B-cell lymphoma patients receiving Bruton tyrosine kinase inhibitors therapy
Source: Front Immunol. 2022 Aug 31;13:982346. doi: 10.3389/fimmu.2022.982346 (PMC9471857; doi:10.3389/fimmu.2022.982346)
Supplement: Supplementary file 1 [file Table_1.docx]

**Supplementary Table 1. Univariate and multivariate Cox regression analysis for OS（N=55）.**

|  | OS (univariate) | | OS (multivariate) | |
| --- | --- | --- | --- | --- |
|  | Crude HR  (95% CI) | *P-*value | Adjusted HR  (95% CI) | *P-*value |
| Female | 0.699  (0.268-1.822) | 0.464 | 1.028  (0.372-2.840) | 0.958 |
| Age﹥60 years | 0.573  (0.228-1.437) | 0.235 | 0.331  (0.120-0.914) | **0.033** |
| IPI（high risk） | 2.779  (1.065-7.251) | **0.037** | 4.258  (1.487-12.191) | **0.007** |
| HBV reactivation | 2.217（0.292-16.841） | 0.442 |  |  |
| chronic and resolved HBV infection | 1.427（0.580-3.514） | 0.439 |  |  |

OS, overall survival; HR, Hazard Ratio; CI, confidence interval; IPI, International Prognostic Index; HBV, Hepatitis B virus.

The significant P-values were bold in this table.
